# Health coaching in primary care: a feasibility model for diabetes care

**DOI:** 10.1186/1471-2296-15-60

**Published:** 2014-04-03

**Authors:** Clare Liddy, Sharon Johnston, Kate Nash, Natalie Ward, Hannah Irving

**Affiliations:** 1 C.T. Lamont Primary Health Care Research Centre, Bruyère Research Institute, 43 Bruyere St., Annex E, Ottawa, ON K1N 5C8, Canada; 2 Department of Family Medicine, University of Ottawa, 43 Bruyere St., Annex E, Ottawa, ON K1N 5C8, Canada; 3 Department of Sociology and Anthropology, University of Ottawa, Ottawa, Canada

## Abstract

**Background:**

Health coaching is a new intervention offering a one-on-one focused self-management support program. This study implemented a health coaching pilot in primary care clinics in Eastern Ontario, Canada to evaluate the feasibility and acceptability of integrating health coaching into primary care for patients who were either at risk for or diagnosed with diabetes.

**Methods:**

We implemented health coaching in three primary care practices. Patients with diabetes were offered six months of support from their health coach, including an initial face-to-face meeting and follow-up by email, telephone, or face-to-face according to patient preference. Feasibility was assessed through provider focus groups and qualitative data analysis methods.

**Results:**

All three sites were able to implement the program. A number of themes emerged from the focus groups, including the importance of physician buy-in, wide variation in understanding and implementing of the health coach role, the significant impact of different systems of team communication, and the significant effect of organizational structure and patient readiness on Health coaches’ capacity to perform their role.

**Conclusions:**

It is feasible to implement health coaching as an integrated program within small primary care clinics in Canada without adding additional resources into the daily practice. Practices should review their organizational and communication processes to ensure optimal support for health coaches if considering implementing this intervention.

## Background

By the end of the next decade, 3.7 million Canadians will live with diabetes [1]. Good glycemic control can decrease the human cost of cardiovascular complications and improve the patient’s quality of life, especially if adopted promptly. It can also help to reduce the long-term economic burden of diabetes on individuals, families, and the health system [1].

Achieving optimal glycemic control demands changes in patients’ health behavior in addition to pharmacological management. This can be challenging for many patients, as recent studies show that 40% of people with diabetes do not reach a target HbA1c of < 7 [2,3]. Most patients require some support to make the necessary behavioral changes [4,5]. Diabetes education can provide patients with this support through information and self-management guidance. A systematic review of diabetes education had mixed results, but concluded that education delivered by a team of educators with *some form* of self-management support follow-up provided the best patient outcomes [6].

### Programs vary

Diabetes education programs vary in the extent to which they adopt a self-management approach. Some self-management support systems are based on self-efficacy theory and aim to provide individuals with the confidence and tools they need to set and achieve health behavioral goals [7]. An example of this type of system is the Stanford Chronic Disease Self-Management Program (CDSMP) [8]. The most widely evaluated self-management program in use, the CDSMP has shown promising outcomes in improved glycemic control, self-efficacy, and health behaviors, especially where glycemic control is poor [9-12]. Studies of other diabetes self-management education programs have also shown positive effects in terms of glycemic control, self-efficacy, and health behaviors [13-15]. However, group programs are not attractive to everyone and such factors as timing, accessibility, literacy level, and transport costs can act as barriers to attendance. Moreover, not everyone who is referred to a diabetes education program attends it, [16] and attrition rates from diabetes education services vary from 4% to 57% [17].

### Health coaching

Health coaching is an intervention offering a one-on-one focused self-management support program described by Lindner et al. as “an interactive role undertaken by a peer or professional to support a patient to be an active participant in the self-management of chronic illness” [18].

As health coaching is a newer intervention, it has not been as widely evaluated as a group program. However, a recent review of 15 randomized health coaching interventions found that six were able to demonstrate significant improvements in one or more health behaviors, including nutrition, physical activity, weight management, and medication adherence [19]. Studies have also demonstrated that health coaching was associated with significant reductions in diabetics’ HbA1c levels [20-22] and weight [23]. Notably, a recent randomized controlled trial of peer health coaching for low-income patients with diabetes demonstrated a 1.07% HbA1c reduction in the coached group, compared with a 0.3% reduction in the usual care group [24]. To our knowledge, health coaching has not been tested in the Canadian context.

We sought an approach to self-management support in primary care that allowed for greater flexibility than the established Chronic Disease Self-Management Program model and had the capacity to tailor the intervention to patients’ needs as they evolved over time. We implemented a health coaching pilot in primary care clinics in Eastern Ontario, Canada and evaluated the feasibility and acceptability of integrating health coaching into primary care for patients who were at risk for or had been diagnosed with diabetes. The purpose of this paper is to report on the feasibility of implementing a health coaching program in a primary care setting. Our lessons learned will be relevant for primary and community-based care organizations, as these groups seek to improve patients’ ability to manage their chronic illnesses.

## Methods

We implemented the program into a purposeful sample of three primary care clinics in Ottawa, Canada in the spring of 2012. The region, which borders on Quebec, has a population of 1.8 million people, approximately half of whom live in Ottawa [25]. Two-thirds of the people living in Ottawa report their mother tongue to be English, 17% French, and 20% a language other than English or French. Twenty per cent of the population are visible minorities. A 2009 report by the Champlain Local Health Integration Network, an organization which serves the Ottawa region, found that 7.5% of individuals over 20 were diagnosed with diabetes in 2005, up from 4.7% in 1996.

The practices were identified because they met three criteria:Two of the sites were academic Family Health Teams that had expressed an interest in participating in this pilot at the proposal writing stage. The third site, a Community Health Centre, was identified through our existing networks and expressed an interest in testing health coaching with an immigrant population (Somalian).

1) They offered team-based care for patients living with chronic disease.

2) Taken together, the patient population of the practices was representative of the general population in terms of language, culture, and social need.

3) There was a sufficient difference in the organization of care in the three practices to gauge feasibility (within the budget and scope of the pilot project).

Ethical approval to carry out this project was granted by the ethics boards of the Public Health Agency of Canada, the Ottawa Hospital, and Bruyère Continuing Care.

### Intervention

Clinic staff were invited to attend a lunch-and-learn session to provide some background to the health coaching project, to discuss the possible impact on the practice, and to answer any questions from team members. Two to three individuals from each site were chosen to be trained as health coaches in the “Peers for Progress” model, [26] which lists four key functions of the health coach:

i) Assistance in applying disease management in daily life (e.g. goal setting, skill building, practice and rehearsal of behaviors, troubleshooting, and problem solving)

ii) Emotional and social support

iii) Linkage to clinical care

iv) Ongoing support

We selected this approach, which is based on the Transtheoretical Model of behavior change, [27] as it is widely used in Motivational Interviewing and fits well with the longitudinal nature of the health coaching role in primary care practices. Coaches received 11 hours of training from expert trainer Amireh Ghorob of the University of California San Francisco’s Centre of Excellence in Primary Care [28]. The curriculum provided practical coaching skills for health care professionals working with patients with diabetes. It trained coaches to assess patients’ confidence in their ability to set and achieve health-related goals using a confidence scale. Coaches were instructed to recognize that if a patient is not ready to change, their role is to provide information and support and revisit goal setting at a later encounter. Coaches were also trained to use Motivational Interviewing [29] techniques such as open-ended questions, and to present a non-judgmental attitude in order to allow patients to explore their current health behaviors and think about change. Eight coaches completed the training. Of this group, one declined participation in health coaching and another was unable to participate. Of the remaining six coaches, three had prior training in motivational interviewing and three were certified diabetes educators.

Health coaching, whether by a peer or a professional, recognizes that self-management education alone is not enough to support individuals with diabetes to sustain behavioral change over a lifetime [30]. While social networks play an important role in supporting individuals to manage their chronic conditions, there are situations where professionals can provide substitute support, or indeed link the patient to community networks [31]. Thus the coaches’ role was to work with referred patients to promote healthy living and improved disease control based on the patients’ goals and state of readiness to change.

We used professional rather than peer coaches because there was no precedent of using peer coaches in a one-on-one setting in the region. This pilot intervention gauged the acceptability of health coaching to patients and the feasibility of implementing coaching in primary care as a first step towards the introduction of peer coaching.

### Patient recruitment

Consenting physicians and nurse practitioners were asked to identify patients (over the age of 18) who had or were at risk of diabetes and who they considered would benefit from health coaching. Teams were specifically asked to identify patients who had not attended diabetes education or group self-management programs: in other words, the hard to reach population who may lack other forms of support. Patients needed to be sufficiently proficient in English, French, or Somali to communicate with their health coach, though literacy in these languages was not mandatory. There were no other exclusion criteria. Identified patients were referred to the program and those who were interested enrolled in the study. Participating patients were asked to provide written or oral consent and invited to complete a baseline survey. No incentives were offered to participate in the study.

Each patient had access to six months of support from their health coach. After an initial face-to-face meeting, follow-up could be by email, telephone, face-to-face, or a mixture of all three. Meetings lasted 30-60 minutes, depending on the coach’s schedule. The suggested time between follow-up meetings was two weeks. Coaches were also encouraged to attend their patients’ regular diabetes visits if work flow practices permitted and the patient and physician/nurse practitioner agreed. Participating sites implemented health coaching to the extent that their staffing and resources allowed. As such, the precise manner of implementation was determined by each site’s existing processes and capacity to adapt to the requirements of a health coaching program.

### Evaluation

We used a mixed methods evaluation [32] and collected data from both practice and patient perspectives using a variety of measures, including the provider focus groups (reported here), and patient surveys and interviews (reported elsewhere).

Two focus groups were held, the first in December 2012 and the second in March 2013. Sessions were facilitated by an experienced qualitative researcher using a topic guide. The project assistant was also available to assist with administration. Health coaches were familiar with one another at the time of the focus groups as they had trained together and in some cases worked together at the same clinic.

Focus groups were chosen to enable an in-depth discussion with the health coaches, which could lead to a greater understanding of the attitudes and perspectives of the coaches and to enable a sharing of thoughts across the groups in order to stimulate further discussions [33]. Focus groups allow researchers to assess participants’ thoughts and the rationale behind them, informing both the micro level interactions and experiences and macro elements, like organizational context [34]. This was particularly relevant to our assessment of feasibility, as we anticipated this factor would be determined not only by their colleagues and patients, but also by the organizational context in which they worked.

### Qualitative data analysis

Qualitative data analysis was conducted throughout the course of data collection. All focus groups were audio recorded and professionally transcribed. Data was prepared for analysis by cleaning and blinding all transcripts. All team members (CL, SJ, HI, KN, NW) reviewed the transcripts to improve rigor and reduce bias. The research team employed a thematic analysis approach, relying on the data-driven nature of its coding to facilitate our study of the feasibility of implementing health coaching into practice [35]. Themes and patterns were identified using open approaches to coding, in which the analysis team worked without preconceived categories, allowing the categories to emerge from the data [36]. The technique of constant comparison [37] was employed to iteratively compare and sort the themes into broad, interrelated conceptual categories. The analysis team met on six occasions over five months to discuss patterns emerging from the data and to evaluate and consolidate themes. Data were then further reduced for analysis using a descriptive matrix, following methods proposed by Miles and Huberman [38]. These matrices were assessed by members of the analysis team who compared them with the initial transcripts to ensure accuracy. Each matrix was organized by theme (barriers, benefits to patients, diversity in coaching method) and contained all data pertaining to that theme. Initially, the matrices were organized and analyzed on a practice-by-practice basis to preserve the analysis team’s ability to assess each practice as a case unto itself [37,39]. This allowed the team to consider negative cases [40] and to better identify similarities and differences across practices, acknowledging the role organizational structure plays in the development and operationalization of professional roles. Once a clear picture of each individual practice emerged, the research team compiled the results in a single matrix to discuss key themes as they related to our research questions.

## Results

All three practice sites were able to implement health coaching using their existing resources. Forty-six patients consented to take part, six of whom did not complete the program. Reasons for declining participation included returning to country of origin, depression, and confusion regarding appointments. Please see Additional file 1 for more information on patient characteristics.

### Focus groups

Two focus groups were held with the six health coaches and two site coordinators. All three sites were represented. The health coaches reported that they successfully implemented the program and felt they had achieved the goals of the study, though their experiences with the process of implementation varied widely. Coaches encountered some common challenges and some difficulties specific to their site. A number of themes emerged, including the importance of physician buy-in, the wide variation in understanding and implementing the health coach role, the significant impact that different systems of team communication had on the role of the health coach, and the significant effect of a clinic’s organizational structure and a patient’s readiness on the health coaches’ capacity to perform their role (see Table 1).

**Table 1:** Explanatory quotes from focus groups with health coaches

**Theme**	**Explanatory quotes**
Physician buy-in	“…I think quite a few physicians were ready, and they did refer their clients. They just gave it (their support) freely. ‘oh I spoke to the patient, and I think they would (be a good candidate for health coaching). Can you please get hold of them?” (Quote 1) – FG2, Nurse 1 (C)
	“I found that generally there wasn’t a lot of support for it (health coaching) (from the physicians)”. (Quote 2) – FG1, Nurse 2 (L)
Health coach role	“I think personally it is hard for the nurse if you run a regular clinic, to be able to take that extra time, to do on top of that the health coaching”. (Quote 3) – FG2, Nurse 1 (C)
	“Being a dietitian, sometimes people were just wondering, “Oh how come you’re doing a medication review?’ Some tasks people were not used to see a dietitian doing. But it’s part of coaching….” (Quote 4) – FG1, Dietician 1 (S)
Communication with the health care team	“..I think having everybody understand what is a health coach about, why it’s so important, I think that would be very beneficial in the future”. (Quote 5) – FG2, Nurse 1 (C)
	“…I still have to go to the doctor and say, ‘What is the target for this patient, what do you want to achieve with this patient?” So for future (health coaching) programs, I wish we had a standard way of communication”. (Quote 6) – FG2, Nurse 1 (C)
	“ I was always emailing back the nurse and the GP so they know what is going on. Even if it was minor, they seemed really pleased to know what was happening”. (Quote 7) – FG1, Dietician 1 (S)
Health coach skills	“And my finding, I was really, really surprised, is what I got the most from the coaching with the patient is just getting to know them, and not so much focussing on the small goals, and …working with the confidence, with the barrier, and you know using a little bit like the stages of change”. (Quote 8) – FG1, Dietician 1 (S)
	“I find the challenge for me is I’m not a dietitian, so I’m being asked to talk about food, etc. etc.” (Quote 9) – FG1, Nurse 2 (L)
	"And what I've been doing, I've been doing with the health coaching, indirectly, using the model I've learned in the program, with the new clients, new diagnoses, and also, also some patients with long-term diagnoses but also who were not really getting involved, who are not taking responsibility of their own health. I was more trying to change my approach. And I would say 95% of the time, it did work well, and you could see people getting more, you know, understanding how important it is to take care of themselves". (Quote 10) – FG2, Nurse 1 (C)
Scheduling follow-up	“I would see a day I wasn’t as busy, and try to fit them in there”. (Quote 11) – FG1, Nurse 3 (N)
Timing and patient readiness	“So once she came here, she was not ready to deal with her diabetes, because she was, she was saying that when you’re depressed you cannot focus on anything else. And so, we had to deal with the depression first, and once we had that stabilized, everything was back to normal she came regularly, saying how helpful (it was)” (Quote 12) - FG2, Nurse 1 (C)
	“I strongly believe that if you want to advocate a bit of well-being, people need to be in power. But do we have the system to be flexible enough so somebody can call and ‘Hey, I’m ready to talk about diabetes now!’ ” (Quote 13) – FG1, Dietician 1 (S)
Benefit to patient	“Her (a participant) doctor was very happy. And haemoglobin A1c is down ….and she (the participant) was saying ‘Thank you,… for the service you guys are doing, but it helped me to see you regularly, to change my lifestyle too”. (Quote 14) – FG1, Nurse 4 (K)
	“I had a patient who none of the numbers changed, but that wasn’t where we were going with this. Just recognizing the relationship between what he eats and what his blood sugars are…It’s like ‘ OK, my blood sugar’s 19, but I went for Chinese food for supper”. OK, so you see that” (Quote 15) – FG1, Nurse 5 (P)
Comprehensive care	“So I found I made a lot more referrals to shared mental health and social worker. And that way they can deal with their depression, and we can work with their diabetes at the same time as their depression is improving, be it through counseling or medication, but so is their diabetes”. (Quote 16) – FG1, Nurse 5 (P)
	“For sure, because they’re being followed with those calls, so I think for sure they’re getting better care, more comprehensive care, because of the follow-up that they wouldn’t otherwise get”. (Quote 17) – FG2, Admin 1
Sustainability	“I think we learned a lot (from the health coaching pilot) and certainly took a lot away from it. And I hope we will be able to implement health coaching in some, in some form at our centre in the next year”. (Quote 18) – FG2, Admin 2

FG1 refers to the first focus group, conducted in December 2012.

FG2 refers to the second focus group, conducted in March 2013.

Support from physicians facilitated recruitment and made implementation of health coaching easier (Quote 1). When this support was lacking, coaches found their role more challenging (Quote 2). While all coaches received the same training, they performed their roles differently depending on their professional position, organizational needs, and interpretation of what a health coach should do. For nurses with an existing heavy workload, incorporating coaching into their routine was challenging (Quote 3). The diabetes nurses and dietitians across sites found it easier to embrace the coaching role (Quote 4), although they noted that other team members did not always understand their role as a coach (Quote 5). Lack of communication with physicians regarding the physician’s goals for the patient was also a frustration (Quote 6). However, health coaching was seen at times as enhancing communication between team members (Quote 7).

Coaches expressed a range of views with respect to the skills employed in coaching. Some coaches focused more on building relationships and less on specific diabetes-related strategies (Quote 8), while others felt that they would have benefitted from specialized, disease-specific knowledge (Quote 9). The strategies presented during training were seen as effective in most cases, especially among patients who were less motivated to change (Quote 10). Following up with patients is an essential part of the coaching intervention, and the ease with which this was achieved varied according to the degree of autonomy coaches had over their schedule. For instance, coaches with little or no autonomy had to be flexible to accommodate follow-up appointments (Quote 11).

Patients’ readiness to change their behavior emerged as a consistent theme, with depression cited as a common barrier (Quote 12). There were situations where patients were dealing with major life events, making it difficult for them to prioritize the management of their diabetes. One coach questioned whether the health care system could be responsive to patients, allowing them to enlist a coach’s services when they were ready (Quote 13). Coaches reported that health coaching had many benefits for participants, including the value of regular support (Quote 14) and improved health literacy (Quote 15). Patients also received more comprehensive care in terms of access to the expertise of the health care team (Quote 16) and the access to support and information when they needed it (Quote 17). For instance, coaches used resources affiliated with the health care team to support patients with depression (Quote 16). In terms of continued sustainability, representatives from all three sites reported that they planned to continue coaching in some form (Quote 18).

## Discussion

Our study found that Canadian primary care providers were able to feasibly integrate health coaching into their primary care clinics without requiring additional resources. Other health coaching programs have had to use additional human resources, such as lay coaches [41] and psychology undergraduates, [22] who do not have other roles within the team. Implementing a health coaching program without these additional resources required considerable commitment from the coaches and a willingness to work around barriers. Despite these challenges, the response from coaches was positive. All three teams expressed an interest in continuing to implement health coaching. However, it is too early to say whether health coaching will become embedded as part of routine practice.

Normalization Process Theory [42] postulates that in order for a new practice to become embedded, actors need to continue to invest in it. Our findings suggest that a successful investment in health coaching has to go beyond the coaches themselves to the whole team. For instance, whether or not physicians supported health coaching had a large impact on the ease or difficulty of its implementation. The importance of physician referral has been noted in other health coaching interventions [21,43]. Focus group discussion revealed that coaches were not always aware of what a physician’s goal was for their patient and found this frustrating. In order to improve sustainability, attention needs to be given to communicating self-management goals through the clinical information systems. This issue has been noted in other studies [20,21]. Chan and colleagues [44] have addressed this concern by building a patient-centric electronic health record to support adherence to lifestyle change, and this is something practices may need to consider to support self-management approaches such as health coaching.

Coaches expressed concern that other members of the health care team were not always aware of their role. In a study involving interviews with health coaches, Wolever commented that coaching is a complex role and needs to be understood by the whole team [45]. The need to understand and appreciate others’ professional roles is not unique to health coaching. As Suter [46] suggests, it is a core competency for collaborative practice.

A common theme that emerged from the focus groups related to the practical issues of arranging follow-up. Health coaches who had some degree of autonomy over their own schedules found it easier to arrange follow-up with patients than did coaches without this autonomy. Practices need to address issues of case load when incorporating health coaching [41].

Lastly, practices may need to plan for the longer term. Swieskowski, describing the introduction of health coaching in a clinic with 130 physicians, said that it took two years for health coaching to take hold [47]. However, he further noted that health coaches became: “the lightning rods for improvement and communication in their practices”, benefitting both the practice and the patients they served.

### Limitations

This intervention was implemented only in practices with existing interprofessional teams and carried out by non-physician members of the team. This approach would not be possible in solo or physician-only practices. However, the teams and implementation approaches were unique across each site, indicating that the intervention can be adapted to a range of existing practice patterns involving interprofessional teams. This intervention was deployed in teams which expressed an interest and which had members willing to pursue additional training in health coaching. The results of this program might not be generalizable to most practices, particularly where an initial interest did not exist.

The focus groups used to evaluate the program’s feasibility in the participating practices were only conducted with the health coaches involved and the practice administrator most responsible for implementing the program. Thus we were unable to assess the buy-in or perception of other members of the team, particularly physicians, for whom this study and others reported in the literature indicate buy-in is critical to success. Finally, we did not collect data on the actual time spent by health coaches in working with their patients. This information would be important to collect in future studies to be able to compare the investment of time in supporting the participating patients compared to usual care or another intervention.

## Conclusion

We successfully implemented health coaching in three primary care clinics and demonstrated the feasibility and potential for sustainability of an integrated patient self-management program. Without adding resources to practices, they were able to implement this highly patient-centered approach to caring for patients who had, or were at risk of having, diabetes. Given that the international literature shows significant potential for this intervention to improve outcomes over the long term, health coaching should continue to be implemented on a broader scale with ongoing evaluation focusing on supportive practice patterns and patient outcomes.

### Practice implications

Our advice to clinical teams who are considering implementing a health coaching program includes:

• Think about whom you are training and the way care at your site is organized. Do the health coaches have the skills and autonomy to offer a truly patient-centered approach?

• Do the coaches have optimal communication with the other providers involved in providing integrated care? This is particularly important to consider when deciding whether coaches will work with patients from many providers or only see patients in their team or teamlet.

• Focus on patients who have barriers to accessing other forms of diabetes education and self-management.

• Consider using health coaches as care coordinators/case managers for patients with complex health care needs, as this may facilitate communication across key providers on goals for the patient.

• If instituting health coaching across a practice, you need to prepare carefully. Be sure to consider such factors as processes, job descriptions, referral, documentation and communications policy, and quality control [48]. This will optimize communication and practice autonomy, and improve understanding of the role of the health coach.

## Competing interests

The authors declare that they have no competing interests.

## Authors’ contributions

CL conceived of and designed the study, participated in data analysis, and contributed to the drafting and revisions of the manuscript. SJ contributed to the study design and implementation, data analysis, and manuscript writing. NW participated in both data collection and analysis and contributed in the writing and review of this article. KN and HI contributed to data collection and analysis and revision of the manuscript. All authors read and approved of the final manuscript.

## Pre-publication history

The pre-publication history for this paper can be accessed here:

http://www.biomedcentral.com/1471-2296/15/60/prepub

## Supplementary Material

Additional file 1 Characteristics of patients participating in health coaching program. Click here for file
