# Supplementary material for: Health coaching in primary care: a feasibility model for diabetes care
Source: BMC Fam Pract. 2014 Apr 3;15:60. doi: 10.1186/1471-2296-15-60 (PMC4021256; doi:10.1186/1471-2296-15-60)
Supplement: Additional file 1 — Characteristics of patients participating in health coaching program. [file 1471-2296-15-60-S1.docx]

Additional file 1: Characteristics of patients participating in health coaching program

|  | Number of participants (n) | Percentage of total sample |
| --- | --- | --- |
| Location^a^ |  |  |
| Site 1 | 11 | 24% |
| Site 2 | 14 | 30% |
| Site 3 | 21 | 46% |
| Sex^a^ |  |  |
| Male | 24 | 52% |
| Female | 22 | 48% |
| First Language^a^ |  |  |
| English | 24 | 52% |
| French | 16 | 34% |
| Other | 6 | 13% |
| Medical History^a^ |  |  |
| Diagnosed with diabetes | 39 | 83% |
| Had ≥ 1 chronic condition in addition to diabetes | 23 | 49% |
| Education^b^ |  |  |
| Had some college or university education | 25 | 53% |

^a^ Totals based on 46 patients who consented to take part in the study

^b^ Totals based on 39 patients who completed a written survey at baseline
